# Classification and Special Nutritional Needs of SGA Infants and Neonates of Multiple Pregnancies

**DOI:** 10.3390/nu15122736

**Published:** 2023-06-13

**Authors:** Chrysoula Kosmeri, Vasileios Giapros, Dimitrios Rallis, Foteini Balomenou, Anastasios Serbis, Maria Baltogianni

**Affiliations:** 1 Department of Pediatrics, University Hospital of Ioannina, 455 00 Ioannina, Greece; 2 Neonatal Intensive Care Unit, School of Medicine, University of Ioannina, 455 00 Ioannina, Greece

**Keywords:** nutrition, preterm, small for gestation age, fetal growth restriction, multiple pregnancy

## Abstract

Data regarding the nutritional management of preterm small for gestational age (SGA) infants are scarce. In the recent report of ESPGHAN, the recommended energy for very preterm infants during hospitalization has been increased, yet this may not fit the needs of all preterm infants. It is important to distinguish fetal growth-restricted (FGR) infants from constitutional SGA infants, as well as preterm SGA from preterm AGA infants, since they may have different nutritional needs. Preterm FGR infants, and specifically infants < 29 weeks’ gestation, accumulate nutrient deficits due to intrauterine malnutrition, prematurity, morbidities, delayed initiation of feeding, and feeding intolerance. Therefore, these infants may need more aggressive nutrition for optimal catch-up growth and neurologic development. However, a balance should be kept between optimal and excessive catch-up growth, since the combination of intrauterine malnutrition and excessive postnatal growth has been linked with later adverse metabolic consequences. Furthermore, multiple gestation is often complicated by FGR and prematurity. There is controversy in the definition of FGR in multiple gestations, and it should be noted that FGR in multiple gestation usually differs etiologically from FGR in singletons. The aim of this review is to summarize existing knowledge regarding the nutritional needs of preterm FGR and FGR infants of multiple gestation.

## 1. Introduction

Although there is plenty of guidance for the nutritional management of preterm neonates, studies regarding the feeding of preterm small for gestational age (SGA) infants are scarce. Moreover, the special nutritional needs of preterm infants of multiple pregnancies, often SGA, are also practically missing. The ESPGHAN Committee on Nutrition of preterm infants has recently recommended to manage the nutrition of fetal growth restricted (FGR) or SGA infants in the same way as appropriate for gestational age (AGA) infants due to the paucity of data to propose specific recommendations. However, they also recommended an individualization of intakes [1].

However, preterm SGA infants may have different nutritional needs from preterm AGA infants, since preterm SGA infants often remain SGA at discharge, indicating accumulative nutrient deficits and failure to catch-up [2]. All preterm infants have deficits of important nutrients crossing the placenta mainly during the third trimester such as iron, calcium, vitamin A, and long-chain polyunsaturated fatty acids [3]. A preterm infant that is also FGR has experienced intrauterine malnutrition. Furthermore, comorbidities in the first days of life in preterm FGR infants, such as necrotizing enterocolitis (NEC) or feeding intolerance, often lead to delayed enteral feeding initiation [2,4]. Because of these factors, preterm FGR infants accumulate more nutrient deficits compared to preterm AGA infants. Furthermore, the combination of intrauterine malnutrition and excessive postnatal growth has been linked with metabolic changes and later adverse metabolic consequences in FGR infants [5,6]. All these factors render preterm FGR infants a special population with unique nutritional needs and a need for special nutritional management. 

The two terms SGA and FGR should not be used as synonyms, since they refer to different conditions [7]. Various fetal, placental, maternal and genetic factors regulate intrauterine growth [8,9]. The definition of SGA includes infants with birth weight less than the 10th percentile, or at least two standard deviations below the mean for the infant’s gestational age, based on data derived from a reference population [10,11]. This can be the consequence of a pathological process or can represent constitutionally small fetuses. 

**Figure 1:**
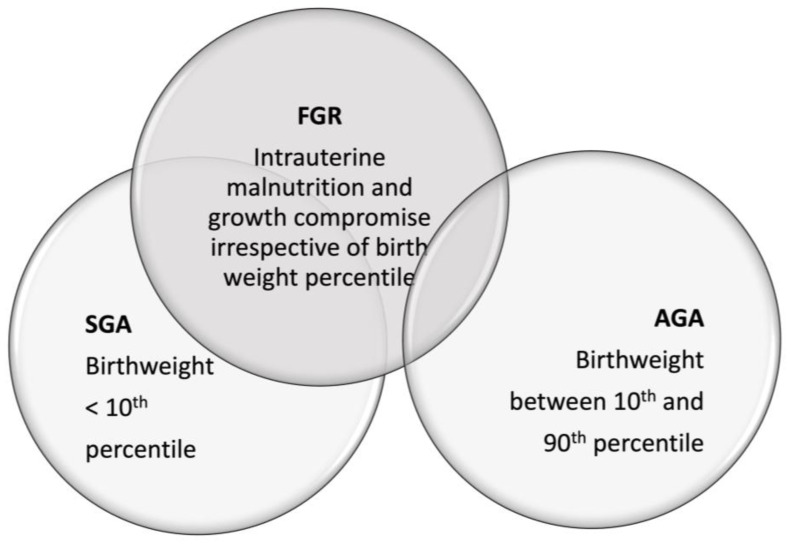
The definitions of small for gestational age (SGA), appropriate for gestational age (AGA) and fetal growth-restricted infants (FGR). Both SGA and AGA infants can be growth restricted.

FGR defines a fetus that fails to reach his potential growth based on race and gender [9]. This term indicates that a neonate has undergone intrauterine malnutrition and growth compromise irrespective of their birth weight percentile [9]. This means that an AGA fetus can be also growth restricted if its intrinsic growth potential was higher [7]. These definitions are shown in Figure 1.

It is challenging to assess the epidemiology of SGA and FGR infants, since some studies use the term SGA to refer to both FGR and constitutionally small infants. The incidence of SGA in well-resourced countries is 10% [12], while in low- and middle-income countries, almost 20% of term infants are SGA [13,14]. A recent study from cities in China found that the overall prevalence of SGA for 2014–2019 was 12.5% for term infants and 7.7% for preterm infants [15]. The prevalence of FGR is higher in resource-limited countries [14] and the incidence increases with decreasing gestational age [16,17]. Moreover, a significant part of all FGR occurs in AGA infants [7]. 

Even though FGR is a common condition, clear guidance for feeding these infants is lacking, and there is still debate on this subject. Studies addressing the optimal way of feeding FGR infants are scarce, and the terms SGA and FGR are often used as synonyms, despite referring to different conditions. Moreover, multiple pregnancies are often complicated with intrauterine restriction, especially when growth charts for singletons are used. This review will focus on existing knowledge and guidance regarding the different nutritional needs of preterm FGR infants and FGR infants of multiple gestation. 

A thorough PubMed and Google Scholar search was conducted using the following terms: preterm infant, FGR, SGA, constitutionally small, multiple gestation, nutrition, and feeding. Relevant studies written in English up to April 2023 were included in this review, and specifically meta-analyses, systematic reviews, clinical trials and observational studies. The reference lists of the articles were also reviewed in the search for other relevant articles that could have been missed in the initial search. The initial search retrieved 420 articles, and after title and abstract screening, a total of 71 articles were used in this narrative review.

## 2. The Distinction between Constitutional SGA and FGR Infants

It is important to distinguish FGR infants from constitutional SGA infants, since these two groups may have different nutritional needs. In the literature, SGA and FGR terms are often used as synonyms based on the idea that a small fetal size is associated with a higher possibility of growth restriction. However, the population is then diluted by healthy SGA fetuses and does not include AGA fetuses that are growth restricted [7]. On retrospective data, it is not possible to distinguish the two categories and exclude mature SGA infants. This is only possible in prospective studies that are designed to detect fetal growth deviations [18]. 

Ananth and Vitzileos hypothesized in their retrospective study that term SGA infants are mostly constitutionally small, while the group of preterm SGA may be comprised mainly by FGR fetuses, since biologic variability is not fully expressed at preterm gestations [19]. Large observational studies suggested that customized growth charts that adjust for the weight, height, ethnicity, and parity of the mother may be better compared to population-based charts in differentiating between constitutional SGA and FGR infants [20,21,22,23]. However, a Cochrane review in 2014 did not find randomized trials to assess the benefits and disadvantages of population-based growth charts compared with customized growth charts [24].

A 2016 consensus definition was established by experts in the field for the antenatal diagnosis of FGR through a Delphi procedure [25]. They concluded that an antenatal diagnosis of FGR includes an abnormal umbilical artery Doppler blood flow profile in addition to reduced growth velocity during fetal life. This means that both SGA and AGA fetuses can be diagnosed as FGR [25]. Furthermore, this consensus allowed for a distinction between early and late-onset FGR.

Therefore, constitutional SGA and FGR terms should not be used as synonyms, and studies in the literature have proposed effective ways to distinguish these two categories of infants.

## 3. Why Do FGR Infants Have Different Nutritional Needs?

It is still unclear what is the optimal nutritional management of FGR infants. On one hand, adequate catch-up growth is essential for the normal neurocognitive development of infants with intrauterine growth restriction [26]. However, excessive catch-up growth in the neonatal period and in infancy has been associated with later adverse cardiovascular and metabolic disorders [5,6,27,28]. A systematic review found an association between rapid infancy weight gain and higher absolute weight-for-length during the first 2 years of life with later obesity [29].

As already mentioned, premature infants have been found to have deficiencies of important nutrients acquired in the third trimester as well as deficiencies in zinc, copper, water- and fat-soluble vitamins and carnitine stores [3]. Infants with FGR have additional nutrient deficits, since they experience intrauterine malnutrition, resulting in chronic growth failure [9].

Because of intrauterine malnutrition, in order to survive, the fetus adapts by redistributing blood flow and nutrients to vital organs, mainly the brain, heart, and adrenal glands, at the expense of other organs. There is also an alteration in the production of placental and fetal hormones that affect fetal growth [9]. This developmental programming can cause epigenetic modifications that occur during a critical period of growth and maturation, resulting in long-term adverse effects. Retrospective studies comparing SGA with AGA infants linked SGA with increased visceral adiposity, increased risk of later hypertension, type 1 and 2 diabetes mellitus, and hyperlipidemia [5,27,28,30]. In multivariate analysis of a retrospective study, FGR was a significant risk factor for increased systolic blood pressure, while prematurity was not. FGR was also associated with reduced renal functional reserve, while prematurity alone was not [5]. A recent meta-analysis of 28 individual studies found that SGA children and adolescents had a 2.33-fold higher risk of type 2 diabetes [6]. 

In addition to these prenatal modifications, the excessive caloric intake after birth and a sedentary lifestyle later in life are associated with increased risk for metabolic syndrome, insulin resistance, and cardiovascular disease [31]. FGR infants can have increased appetite because they lack satiety due to an imbalance of their hypothalamic orexigenic/anorexigenic neuropeptides [9]. This may be beneficial leading to catch-up growth, but it may also contribute to later adverse metabolic effects.

Therefore, FGR infants may need special nutritional management, since neither iatrogenic malnutrition and insufficient catch-up growth nor excessive fat tissue gain are acceptable practices. Furthermore, FGR infants may accumulate nutrient deficits in the early postnatal life due to the higher risk of morbidities, the need for hospitalization, and the hesitation to start early feeding or the intolerance of feeding [4,32]. This is more prominent in preterm FGR infants. 

One goal when feeding these infants is the administration of sufficient nutrients so postnatal growth would be similar to that of a normal fetus of the same gestational age or an infant with the same postmenstrual age. The intrauterine fetal growth is at least 15–20 g/kg/day. Tudehope et al. proposed in their review that a caloric intake of at least 110–135 kcal/kg/day with additional energy may be necessary for SGA infants for adequate catch-up growth to reach the rate of in utero growth [33].

On the other hand, constitutional SGA infants do not experience intrauterine malnutrition; therefore, they may have different metabolic needs. There are no studies focusing mainly on constitutional SGA infants except a prospective study of 58 constitutional SGA infants which found no increased risk for metabolic sequalae in the first 24 months of age [34]. Therefore, constitutional SGA infants should receive normal newborn care [33], although more prospective studies are needed.

## 4. The SGA Infant and Metabolic Consequences

The combination of poor fetal and accelerated postnatal growth rates in SGA infants appears to act in synergy in later metabolic adverse events [31]. In most SGA infants, catch-up growth occurs in the first 2 years of life and mostly in the first 6 months [35,36,37].

A systematic review of two randomized trials of term SGA infants concluded that enriched infant formulas that promoted early growth and contained 28–43% more protein and 6–12% more energy than the control formula increased fat mass, lean mass, and blood pressure at the age 5–8 years [38]. The authors of the two RCTs recommended that higher caloric intake and rapid weight gain are not optimal for these children, and therefore, breastfeeding should be preferred, since it was associated with slower weight and height gain [39,40]. However, a more recent systematic review of term SGA infants showed that children receiving exclusive breastfeeding had no body composition alteration or increased insulin resistance compared to children receiving a higher calorie formula [41]. Therefore, it seems that breastfeeding should be preferred over enriched infant formulas in term SGA infants. 

Prospective and observational studies of term SGA infants showed that rapid weight gain and length catch-up growth during early postnatal life, specifically in the first 3 months of life, were associated with lower insulin sensitivity, lower HDL-cholesterol concentrations, higher triglyceride concentrations, obesity, and markers of atherosclerosis in early adulthood [42,43]. It is worth mentioning that these studies did not distinguish between constitutional SGA infants and FGR infants. Furthermore, it is important to note that these studies refer to term SGA infants, while similar data on preterm SGA infants are lacking. Therefore, it is unclear whether these findings apply to preterm SGA infants as well. 

A recent systematic review and meta-analysis that included either preterm or SGA infants found that early macronutrient supplementation led to greater weight and length in toddlers without an increase in the risk for metabolic disease later in life. The data were mostly for toddlers and older children, while data for older ages were limited [44]. In a subgroup analysis, this study also found that supplementation decreased triglyceride concentrations but increased cholesterol concentrations at age older than 3 years and increased fasting insulin concentrations in adolescence only in SGA and not in AGA children. However, due to the small sample, since the three trials included only 25 SGA children, a safe conclusion about the effects of macronutrient supplementation on SGA infants could not be drawn [44].

Therefore, data indicate that term SGA infants may experience later adverse metabolic effects after excessive catch-up growth. However, available studies did not distinguish between FGR and constitutional small infants, and there is a paucity of data regarding the possible metabolic consequences in preterm FGR. Thus, future prospective and properly designed studies in SGA preterm are necessary to answer this question.

## 5. Fetal Growth Restriction in Multiple Gestation

There is controversy in the definition of FGR in multiple gestations. Twin pregnancies are often complicated with intrauterine growth restriction and prematurity. One or both fetuses can be restricted, and FGR infants have increased morbidity. In the cohort study LEMON of monochorionic diamniotic twins with selective FGR (sFGR), it was found that smaller twins had mild neurodevelopmental impairment compared to the larger twin, while no difference was found in children who had severe neurodevelopmental impairment between the two groups [45]. 

The FGR in singletons usually etiologically differs from FGR in twins, and the definition of FGR in multiple pregnancies is different in the literature. The National Institute for Health and Care Excellence (NICE, 2019) defines sFGR as the discordance of estimated fetal weight (EFW) 25% and above or EFW of one fetus below the 10th centile for gestational age [46]. The International Federation of Gynecology and Obstetrics (FIGO) recommends using twin-specific growth charts to avoid the overdiagnosis of sFGR [47]. 

A 2019 consensus definition was established by experts in the field for the sFGR through a Delphi procedure. The panel agreed that EFW of one twin < 3rd centile, irrespective of chorionicity, was adequate for a definition of sFGR. Moreover, at least two contributory parameters out of the following would define sFGR in monochorionic twin pregnancy: EFW of one twin < 10th centile, abdominal circumference of one twin < 10th centile, EFW discordance of ≥25%, umbilical artery pulsatility index of the smaller twin > 95th centile. In dichorionic twin pregnancy, EFW of one twin < 3rd centile and at least two out of three of the following contributory parameters was agreed to define sFGR: EFW of one twin < 10th centile, EFW discordance of ≥25%, umbilical artery pulsatility index of the smaller twin > 95th centile [48]. The Delphi definition recommends using singleton growth charts to assess fetal growth in twin pregnancies. 

In a case-control study of 313 twin pregnancies, FGR was defined as one twin having a birth weight < 10th or <5th percentile for gestational age or a birth-weight discordance ≥ 20%. In this study, a twin with birth weight < 10th percentile was found in 47% of pregancies, at least one twin with a birth weight < 5th percentile was found in 27% of pregnancies, and in 16% of patients, there was a birth-weight discordance of ≥20% [49]. In these tables, the 10th percentile curve in singletons is similar to the 25th percentile curve in twins, demonstrating that almost 25% of twins are below the 10th percentile for birth weight based on singleton norms [49].

The incidence of FGR in twin pregnancies was reported 15 to 47% in the literature [49,50,51]. Selective FGR was found in 10–15% of all monochorionic pregnancies, and it was a cause of significant perinatal mortality and morbidity [52]. 

There are limited data to guide the management of twins affected by FGR, including optimal nutritional management, whereas the need for prospective nutritional studies seems to be imperative. The nutrition of FGR infants of multiple gestation will be discussed in this review together with the nutrition of preterm FGR infants due to a lack of studies. However, these infants are not similar to preterm FGR infants, since the pathophysiology of small growth may be the consequence of limited intrauterine space and not of intrauterine malnutrition. 

## 6. The Preterm FGR Infant

Preterm and term FGR infants should not be comparable, since they are different regarding their maturity, growth, and nutritional needs. It is unclear whether poor growth, catch-up growth, and early nutrition have similar effects in preterm and term FGR infants [53]. Data regarding the best feeding practices of preterm SGA and specifically preterm FGR infants are lacking. Considerations when feeding the preterm FGR infants are shown in Table 1.

In their recent report, the ESPGHAN authors have increased the recommended energy for very preterm infants during hospitalization [54]. There are some concerns that even this increased amount of energy does not fit the needs of all preterm infants [55,56,57,58]. This may be the case, especially in SGA preterm infants with higher nutritional deficits whose needs may be different than those of preterm AGA infants. As already mentioned, regarding FGR and/or SGA infants, the ESPGHAN committee proposed the same nutritional management as for AGA infants due to the paucity of data, although initial weight loss is often less and acceptable up to 4–7% of birth weight [1,54]. There was no distinction between constitutionally SGA and FGR infants.

The goal Is to optimize enteral nutrition in preterm FGR neonates without increasing the risk of morbidities, such as NEC [2]. FGR infants are at increased risk of NEC due to postnatal impaired gut function secondary to intrauterine malnutrition and a consequent reduction in gut perfusion [4,16,32].

Another question in clinical practice is the optimal timing of starting enteral nutrition, especially in infants with abnormal Doppler studies. In these infants, delayed enteral feeding due to the fear of NEC can lead to additive nutrient deficits. For the immediate postnatal period for preterm SGA, if the abdominal examination is normal, Dutta et al. suggested starting feeding in the first day of life, with slow advancement of volumes at the lowest end of the range [59]. A randomized trial of preterm FGR infants (SGA preterm who had abnormal antenatal umbilical Doppler flows) found that an initiation of minimal enteral feeding in the first 5 days of life or less did not affect the incidence of NEC or feeding intolerance [60]. In another randomized trial of 404 infants younger than 35 weeks’ gestation, it was shown that milk feed initiation on the second day of life led to earlier full milk feed achievement by 3 days compared to feed initiation on day 6, with no difference in NEC incidence. However, the group of preterm FGR infants younger than 29 weeks’ gestation reached full enteral feeds at a median age of 9 days later than predicted from the study feeding regimen, and they also had an over three-fold increased incidence of NEC compared to older infants. Feeding intolerance occurred at a younger age, at lower milk volumes, and lasted longer in the group of infants younger than 29 weeks’ gestation [61]. Similarly no difference in the incidence of NEC was observed in an RCT on preterm SGA infants that received minimal enteral feeding for five days compared to no enteral feeding [62]. Another randomized trial on 133 IUGR infants comparing the effects of an early versus late enteral feeding in preterm FGR infants had the same conclusion, since the onset of feeding did not affect the incidence of NEC or feeding intolerance [63].

Most of these RCTs were included in a recent Cochrane review. This review included 1551 very preterm or very low birth weight infants, half of which were FGR or had reversed end-diastolic flow velocities in the fetal aorta or umbilical artery. They found that there was no reduced risk in NEC or all-cause mortality, but there was only a slightly decreased risk of feed intolerance after the delayed introduction of progressive enteral feeds. However, this was low-certainty evidence [64]. 

Dutta et al. proposed in 2015 that an advancement of feeds should be extremely slow in preterm SGA babies with gestation < 29 weeks and absent/reversed end-diastolic umbilical flow. Specifically, they suggested that an advancement of volumes in the first 10 days of life should be at the lowest end of the range [59]. This is due to the results of a randomized trial that showed that these infants failed to tolerate even a slow advancement of feeds. The median volume of milk tolerated in the first 10 days was <20 mL/kg/day. The median age to reach full feeds (150 mL/kg/day) was 28 days compared to 19 days in the group of infants 29 to 34 + 6 weeks gestational age with abnormal antenatal Doppler studies [65]. This means that infants born < 29 weeks’ gestation may have additional nutrient deficits, and therefore, they may need more aggressive nutrition later to catch-up.

The American Society for Parenteral and Enteral Nutrition guidelines recommended early minimal enteral feeding within the first two days of life and advancement of feeding volumes at 30 mL/kg/d in infants weighing ≥ 1000 g [66]. It remains under question whether this applies also to FGR preterm infants. The strength of the recommendation was weak; therefore, larger multicenter prospective trials are mandatory to answer questions regarding the initiation of enteral feeding and feeding volume advancement. 

Human milk is preferred over formula [59]. In a retrospective study of 420 preterm SGA infants, human milk was associated with a decreased risk of NEC and late-onset sepsis [2]. Even in the very high-risk group of preterm infants < 29 weeks gestation, breast milk was protective against NEC [65]. A Cochrane review in preterm and LBW infants found that formula feeding led to greater weight gain, linear and head growth and a higher risk of NEC compared with donor breast milk. This evidence was of moderate certainty. There was no difference in mortality, long-term growth or neurological development [67].

As already mentioned, preterm FGR infants may accumulate nutrient deficits during the first days of life due to the higher risk of morbidities, the need for hospitalization, and the hesitation to start early feeding or to feeding intolerance. Cohorts of SGA preterm infants showed a high incidence of postnatal growth failure [2,17,68]. In a cohort of 1776 SGA infants, 97% of them remained SGA at 36 weeks’ corrected age [17], and the same was shown in a smaller cohort of 104 preterm infants of whom 21% were SGA and remained SGA at 40 weeks postmenstrual age [68]. Growth restriction in preterm neonates was found to be inversely related to younger gestational age and weight at birth [69]. A small study of preterm SGA infants showed that an exclusive human milk-based diet led to weight gain equal to the AGA group during a follow-up period of 12–15 months with no increased adiposity or elevated insulin resistance at 2 years of age [70]. On the other hand, in a retrospective cohort of 420 preterm SGA infants, growth failure persisted at discharge in infants receiving either exclusive human milk or cow’s milk diet [2]. 

Senterre et al., in their prospective observational study of preterm SGA infants, demonstrated that SGA infants were able to tolerate higher nutritional intakes during the first weeks of life and had higher weight gain compared to AGA. Preterm SGA were started with nutritional intakes of 40 ± 6 kcal/kg/day, while enteral feeds were introduced at a mean age of 2.9 ± 4.2 days. In the third week, the mean nutritional intake was 130 ± 17 kcal/kg/day and the enteral intake was 127 ± 55 mL/kg/day. Parenteral nutrition was discontinued at a mean age of 21.3 days [71]. The authors concluded that nutritional supply in the first days of life is essential to improve growth in the first week of life and to limit postnatal weight loss and growth restriction. 

## 7. Conclusions

Available guidelines did not provide specific recommendations for SGA or FGR infants due to the paucity of studies. Prospective studies are mandatory to assess the optimal nutritional management of different groups of infants. Constitutional SGA infants, FRG infants and specifically preterm FGR and FGR infants of multiple pregnancies should be distinguished in future guidelines, and specific recommendations should be given for each group.

In conclusion, in preterm FGR infants, a balance should be kept between the optimal catch-up growth that is vital for normal neurologic development and excessive catch-up growth that may lead to the development of cardiovascular and metabolic disorders later in life. Preterm FGR infants have accumulative nutrient deficits due to prematurity, fetal malnutrition, comorbidities in the first days of life, delayed initiation of enteral feeding, slow advancement of feeds, and tolerance of lower feeding volumes. This is more evident in infants < 29 weeks’ gestation. This means that they may need more aggressive nutrition later to catch up, since data indicate that many preterm SGA infants remain SGA at discharge. Breastfeeding is preferred over enriched infant formulas to reduce the risk of NEC and later metabolic adverse consequences.

## Figures and Tables

**Table 1:** Considerations in nutritional management of preterm FGR infants.

Considerations in Nutritional Management of Preterm FGR Infants
Optimal catch-up growth is essential for proper neurocognitive development
Excessive catch-up growth may lead to adverse metabolic consequences later in life
Increased nutrient deficits due to intrauterine malnutrition, prematurity, and morbidities in the early postnatal life
Increased risk of NEC due to postnatal impaired gut function secondary to intrauterine malnutrition and a consequent reduction in gut perfusion
Delayed feeding initiation due to fear of NEC
Slow advancement of feeds, more time to reach full enteral feeds due to feeding intolerance
Human milk is preferred over formula, since it was found to be protective against NEC and later adverse metabolic consequences

## Data Availability

Not applicable.
